# Influence of voids in the hybrid layer based on self-etching adhesive systems: a 3-D FE analysis

**DOI:** 10.1590/S1678-77572009000700005

**Published:** 2009

**Authors:** Ana Paula MARTINI, Rodolfo Bruniera ANCHIETA, Eduardo Passos ROCHA, Amilcar Chagas FREITAS, Erika Oliveira de ALMEIDA, Renato Herman SUNDFELD, Marco Antonio LUERSEN

**Affiliations:** 1 Undergraduate student, Dental School of Araçatuba, São Paulo State University-UNESP, Faculty of Dentistry of Araçatuba, SP, Brazil.; 2 DDS, MSc student, Department of Dental Materials and Prosthodontics, Faculty of Dentistry of Araçatuba, São Paulo State University-UNESP, Araçatuba, SP, Brazil.; 3 DDS, MSc, PhD, Assistant Professor, Department of Dental Materials and Prosthodontics, Faculty of Dentistry of Araçatuba, São Paulo State University-UNESP, Araçatuba, SP, Brazil.; 4 DDS, MSc, PhD student, Department of Dental Materials and Prosthodontics, Faculty of Dentistry of Araçatuba, São Paulo State University-UNESP, Araçatuba, SP, Brazil.; 5 PhD, Department of Restorative Dentistry, Faculty of Dentistry of Araçatuba, São Paulo State University-UNESP, Araçatuba, SP, Brazil.; 6 PhD, Department of Mechanical Engineering, Universidade Tecnológica Federal do Paraná-UTFPR, Curitiba, Brazil.

**Keywords:** Hybrid layer, Voids, Dentin, Finite element analysis

## Abstract

The presence of porosities at the dentin/adhesive interface has been observed with the use of new generation dentin bonding systems. These porosities tend to contradict the concept that etching and hybridization processes occur equally and simultaneously. Therefore, the aim of this study was to evaluate the micromechanical behavior of the hybrid layer (HL) with voids based on a self-etching adhesive system using 3-D finite element (FE) analysis. Material and Methods: Three Fe models (Mr) were built: Mr, dentin specimen (41x41x82 μm) with a regular and perfect (i.e. pore-free) HL based on a self-etching adhesive system, restored with composite resin; Mp, similar to M, but containing 25% (v/v) voids in the HL; Mpp, similar to Mr, but containing 50% (v/v) voids in the HL. A tensile load (0.03N) was applied on top of the composite resin. The stress field was obtained by using Ansys Workbench 10.0. The nodes of the base of the specimen were constrained in the x, y and z axes. The maximum principal stress (σ_max_) was obtained for all structures at the dentin/adhesive interface. Results: The Mpp showed the highest peak of σ_max_ in the HL (32.2 MPa), followed by Mp (30 MPa) and Mr (28.4 MPa). The stress concentration in the peritubular dentin was high in all models (120 MPa). All other structures positioned far from voids showed similar increase of stress. Conclusion: Voids incorporated into the HL raised the σ_max_ in this region by 13.5%. This behavior might be responsible for lower bond strengths of self-etching and single-bottle adhesives, as reported in the literature.

## INTRODUCTION

The structural behavior of the adhesive layer plays an important role in maintaining the integrity of the dentin-resin bond over time^1^. Self-etching adhesive systems have been introduced to allow dry bonding based on shallower demineralization with the formation of thinner hybridization of dentin. Consequently, a more homogenous dentin/adhesive interface is expected to be recreated^5^. Self-etching adhesives also reduce the steps necessary for bonding in comparison with etch-and-rinse adhesives. Because many self-etching adhesives leave the bottom of smear plugs intact, they tend to create resin-dentin bonds that exhibit less dentin sensitivity^5^^,^^6^.

Although self-etching adhesives are used in order to form a stable and strong biopolymer^18^, lower bond strength has been reported^4^ mainly in wet enviroments^14^. Unfortunately, the benefit of saving time by using self-etching adhesives may impair the quality of resin–dentin bonds (e.g. incomplete sealing)^6^.

The complex environment of hybrid layers (HL) created by self-etching adhesives may explain their reduced performance^30^. For instance, nano and micro analysis of one-bottle adhesives show the presence of high concentrations of hydrophilic acid monomers as being responsible for the incorporation of voids in these hybrid layers that, in turn, increase their permeability^24^^,^^29^. In these zones, water is incompletely removed, resulting in regions of imperfect polymerization and/or hydrogel formation between the remaining water and the HEMA present in the adhesive systems^7^^,^^25^. Therefore, partial hybridization of dentin may occur with more aggressive self-etching adhesives (lower pH). The presence of these defects may act as stress raisers^19^ in resin-dentin interfaces, reducing adhesion over time.

A previous study provided data about bond strength and the characteristics of the adhesive interface obtained using scanning electron microscope (SEM) and nanoleakage^9^^-^. However, little information is available about the mechanical behavior of the dentin/adhesive interface based on hybrid layer quality^2^^,^^15^^,^^19^.

The aim of this study was to evaluate the mechanic behavior of the hybrid layer containing voids, using 3-D finite element analysis. The voids were incorporated in different proportions (25% and 50% by volume). The null hypothesis is that voids have no effect on stresses within the hybrid layer.

## MATERIAL AND METHODS

In order to perform the micromechanical analysis of dentin/adhesive interfaces, a virtual dentin specimen restored with composite resin^18^(41 x 41 x 82 μm) was built using SolidWorks software (SolidWorks Corporation, Concord, MA, USA)^2^. The dimensions of each structure of the model and their mechanical properties were based on previous data, assuming a linear, isotropic and linearly elastic study (Table 1).

**Table 1:** Dimensions (μm) and mechanical properties of the materials (E and ν)

Structures		Dimension (μm)	*E* (GPa)^18^	ν^15^
Specimen	Width (base)	41x 41		
	Length	82		
Composite resin		41	30	0.3
Adhesive layer^18^		2 (Length)	5	0.28
Hybrid layer^26^		4 (Length)	4	
			3	0.28
			2	
			1	
Intertubular dentin close to HL^2^^,^^18^		3 (Length)	13	0.3
Intact intertubular dentin^18^		36 (Width)	20	0.3
Diameter of Peritubular dentin^18^		0.75 ( Width)	28.6	0.3
Pulp^17^			0.0002	0.45
Resin Tag^26^		17 (Length)	5	0.28
Number of dentinal tubules^8^		16 (deep dentin)		
Diameter of dentinal tubules^20^		1 (deep dentin)		
Diameter of spherical voids		3.75		

**Figure 1:**
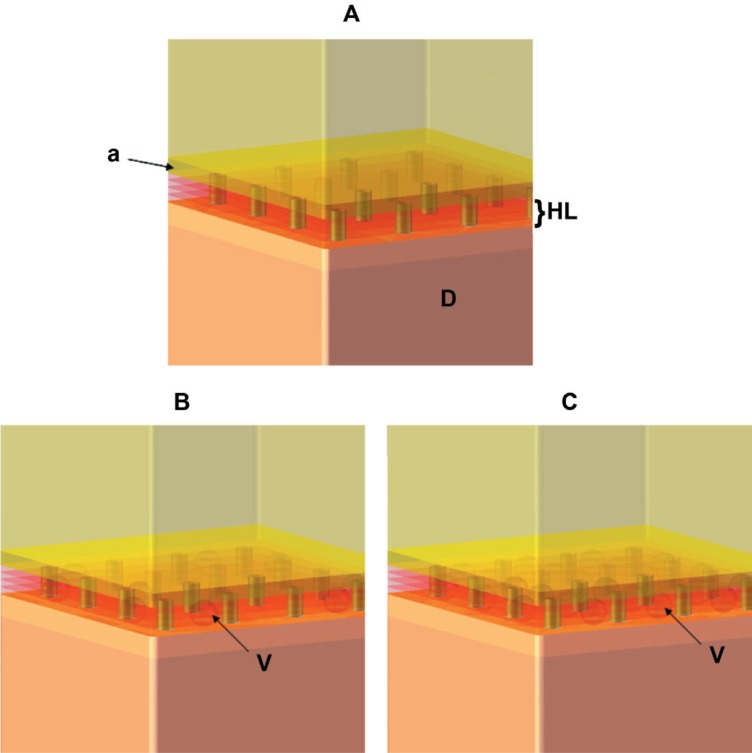
A - Model (Mr) with perfect bond between the hybrid layer (HL) and dentin (D); B – Model (Mp) with 25% of voids (V) in volume in the HL; C – Model (Mpp) with 50% of voids in volume in the HL. (Diameter of voids = 3.75 μm). HL (hybrid layer); V (voids); D (dentin); a (adhesive layer)

Considering the dimensions reported in Table 1^8^^,^^15^^,^^17^^,^^18^^,^^26^, three models were built by varying their void content in the HL by 0%, 25% or 50% (Mr, Mp, Mpp, respectively) (Table 2, Figure 1). Mr represents a perfect HL, without voids and completely infiltrated. Mp and Mpp represent a HL containing 25% and 50% of the volume with voids, respectively.

**Table 2:** Percentage of voids in the hybrid layer

Models	% of voids in the HL
Mr	0 (ideal bond)
Mp	25
Mpp	50

It was assumed that the adhesive that infiltrated through the collagen fibrils in these models bonded to adjacent and subjacent structures. Regarding the rigidity of the bonded structures, Misra, et al.^18^ (2004) established that the elastic modulus of peritubular dentin and intertubular dentin show values of 28.6 GPa and 20 GPa respectively. However, Katz, et al.^12^ (2001) reported that the elastic modulus of the intertubular dentin, adjacent to the HL, has a value of 13 GPa, due to effect of previous etching required by the conventional adhesive system. Unfortunately, these data have not been reported for aggressive self-etching adhesive systems.

In their study, Misra, et al.^18^ (2004) varied the HL thickness between 2 and 10 μm. These authors considered the HL to be constituted of several sublayers of equal thickness. Thus, the elastic modulus was graded from 4 GPa for the most superficial sublayer in contact with the adhesive layer, to 1 GPa for the deepest sublayer in contact with the mineralized dentin base. According to these authors, due to the lower dimethacrylate adhesive infiltration of deep demineralized dentin, hybrid layers may be built with different elastic modulus in order to reproduce *in vivo* conditions. In the present study, a 4 μm thick HL was stratified in four 1 μm thick layers. A 4 GPa elastic modulus was used for the layer closest to the adhesive material; 3 GPa for the second layer, 2 GPa for the third layer and 1 GPa for the deepest layer in contact with the adjacent mineralized intertubular dentin^18^.

The convergence criterion was applied to reach optimal mesh quality. All models showed up to 38.497 tetragonal elements and 102.580 nodes. The nodes at the base of the specimen were fixed on the x, y and z axes (x=y=z=0) to set up the border line.

In order to determine the loading value, it was observed that in a dentin macro-specimen (5x2x2 mm) restored with composite resin, in an hourglass shape (sectional area of 1.1 mm^2^) there was an equivalent tensile force of 18 MPa after 20 N of tensile loading at the top of the resin surface, exactly as previously described^22^.

**Figure 2:**
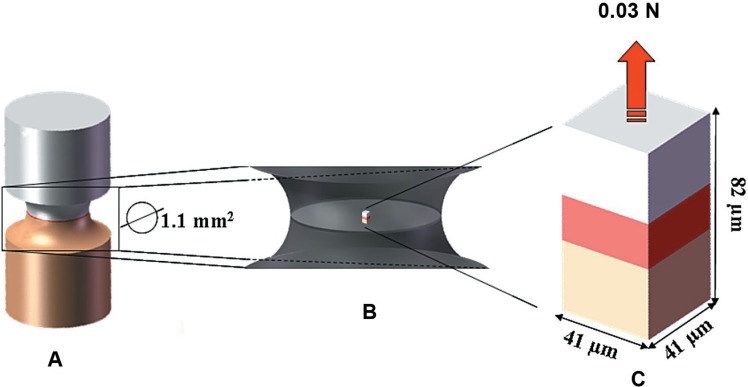
A - Hourglass-shaped specimen with 1.1mm^2^ of sectional area; B - Visualization of the model inside the hourglass-shaped specimen; C - High magnification of the model. Model dimensions (41x41x82 μm) and loading condition (tensile load, perpendicular to the top of the composite resin, with 0.03 N)

The cross-sectional area of the micro-specimen model in the present study was equal to 1681 μm^2^. To apply an 18 MPa tensile force to the adhesive interface, a tensile load equal to 0.03N was perpendicularly applied to the composite resin surface (Figure 2).

As developed by Misra, et al.^18^ (2004), the maximum principal stress (σ_max_) was utilized for identifying failures that could start out of small flaws, and it is an adequate criterion for brittle structures, such as dentin. All structures at the interface, i.e. peritubular dentin, intertubular dentin, adhesive layer and HL, were individually analyzed.

The numerical analysis was performed using ANSYS Workbench 10.0 (Swanson Analysis System, Canonsburg, PA, USA).

## RESULTS

**Figure 3:**
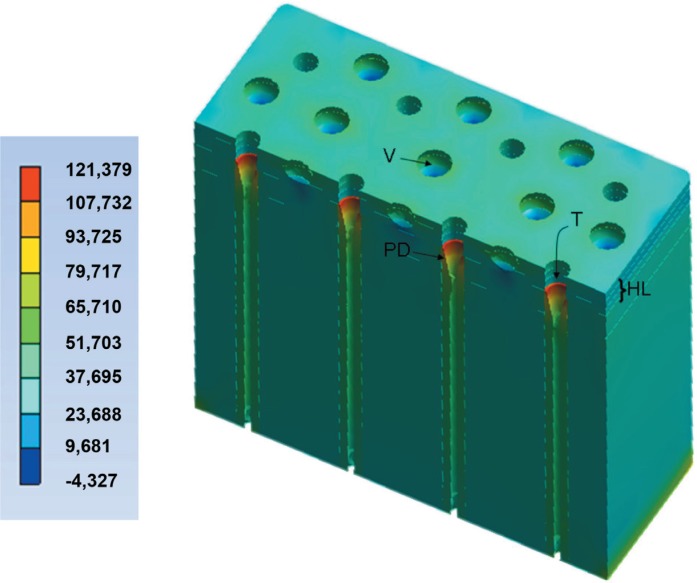
Maximum principal stress (σ_max_) in the peritubular dentin (PD) and void (V) for the Mpp. Stress concentration in the three deepest layers of the hybrid layer (HL), voids (V), peritubular dentin (PD) and dentinal tubules (T) with no resin tags

**Figure 4:**
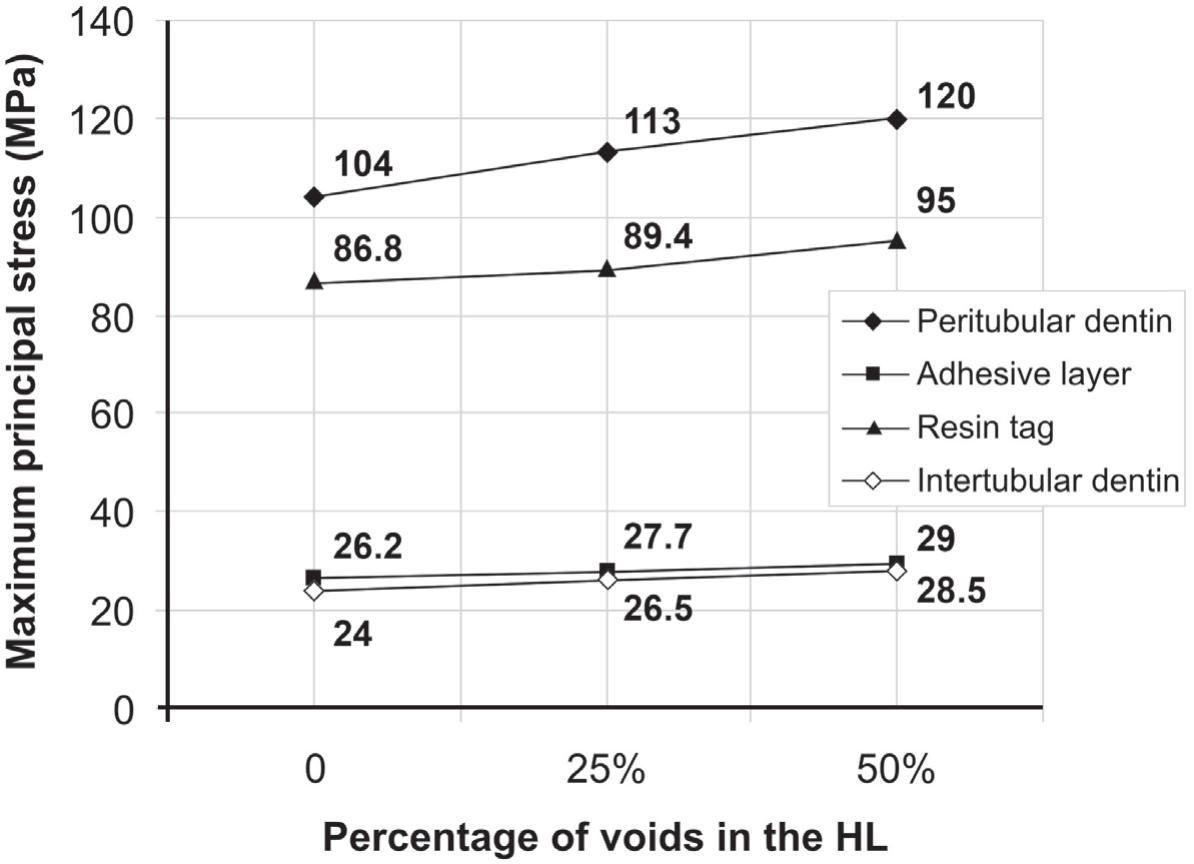
Maximum principal stress (MPa) in the peritubular dentin, adhesive layer, resin tags and intertubular dentin according to the percentage of voids (0, 25% and 50%) in the hybrid layer

**Figure 5:**
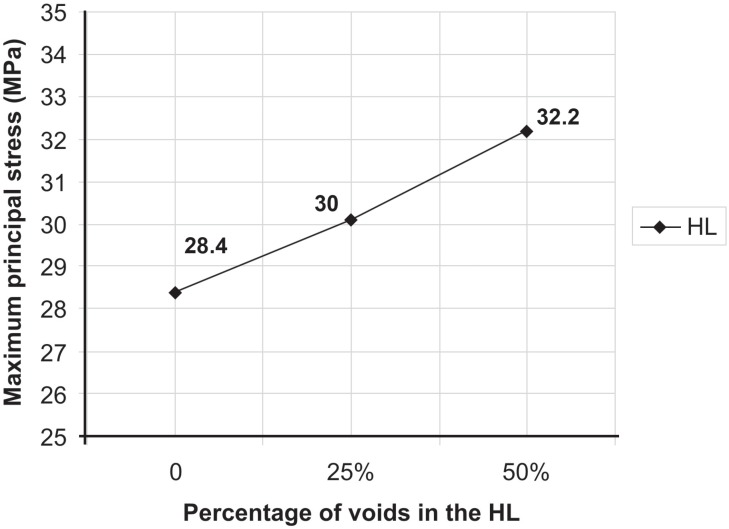
Maximum principal stress (MPa) in the hybrid layer according to the percentage of voids (0, 25% and 50%) for Mr, Mp and Mpp, respectively

The peak of σ_max_ was observed in the peritubular dentin (Figure 3) for all models (Figure 4). This behavior was similar in all other structures of the dentin/adhesive interface that showed an increase of σ_max_ in Mp when compared with Mr and in Mpp when compared with Mp (Figure 4 and 5). Figure 4 shows the σ_max_ for the adhesive layer, peritubular dentin, resin tags and intertubular dentin.

**Figure 6:**
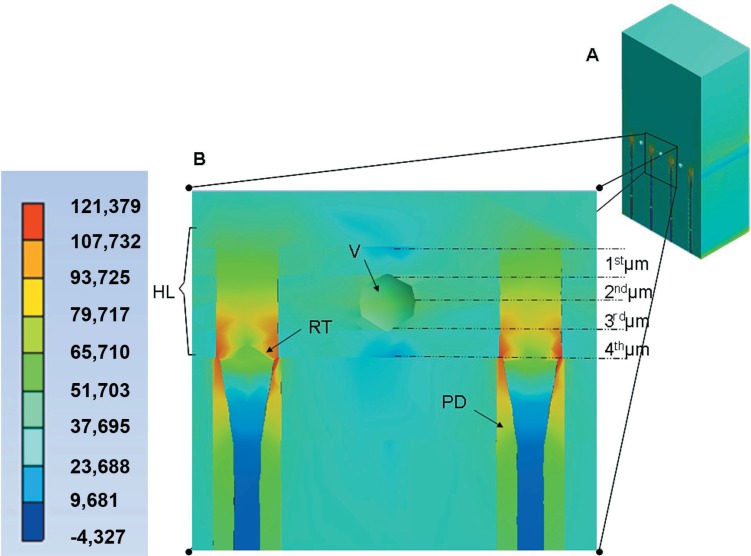
Maximum principal stress (MPa) for the Mpp. A – Cross-section view of the model Mpp to observe stress distribution (high magnification in B). B - Stress concentration on top of resin tags (RT), peritubular dentin (PD) and in lower intensity in the porosities (V). Note that the sectioning was done to visualize half of the dentinal tubules. This allowed partial visualization of porosities

The σ_max_ in the peritubular dentin was observed in its upper border, exactly where the resin tags start (Figure 3). The σ_max_ in the tags occurred in their upper border (top of tags), in contact with the dentinal tubule walls (Figure 6).

The increase in the percentage of porosities within the HL, like in other structures, negatively influenced its mechanical behavior, increasing the stress concentration (Figure 5). The increase in stress in the HL was of 6% in Mp when compared with Mr and of 6.5% in Mpp when compared with Mp.

The σ_max_ in the HL occurred near the porosities and between the second and third micrometers deep in the Mp and Mpp (Figure 6).

## DISCUSSION

While a durable seal between several current bonding systems and enamel has been achieved, it is still a challenge to seal the resin-dentin interface, due to the heterogeneous characteristic of the dentin structure, surface morphology^10^, and/or intrinsic shortcomings of the design of these modern adhesives^21^.

Conventional thought is that a perfect seal along the resin-dentin interface can be achieved within the demineralized collagen matrix when it is completely infiltrated by adhesive resins in permanent and primary teeth^11^. This concept is based on the assumption that the polymerized resins used for bonding are nonporous and impermeable to fluids^23^. However, adhesive phase separation into hybrid and adhesive layers do not create an impervious collagen/polymer network but instead produce a porous web, and at the same time, affecting the chemical and mechanical properties of the adhesive layer^27^.

Consequently, in order to evaluate the influence of the HL voids on the stress distribution at dentin/adhesive interfaces in this study, the bond established between the adhesive system and dentin was either considered ideal or incorporated with voids in different contents (25% and 50%).

It was observed that the peritubular dentin showed the highest stresses in all models, in accordance with previous studies^2^^,^^3^. The next structures to bear high stress were the resin tags, HL, adhesive layer and intertubular dentin.

The increased stress concentration observed in the top of tags can be related to the dentinal tubule diameter (1.0 μm) and closeness to the peritubular dentin which shows the highest elasticity modulus among the structures simulated at the dentin/adhesive interface^2^^,^^18^.

The voids in the HL (Mp and Mpp) increased the σ_max_ to values 13.5% higher in comparison with the void-free perfect HL (Mr). Nevertheless, the σ_max_ in the 3 models were below the failure load established for the adhesives^28^. This indicates that the bottom of the adhesive layer might not be the site to start up failure when the bond between dentin and HL is porous. The peak of σ_max_ in the Mr was in the HL close to the peritubular dentin, very similar to that previously found in a perfect bond scenario^2^^,^^3^.

Mollica, et al.^19^ found that the presence of voids raised the σ_max_ by 3.7 times. In our study, the influence of voids was able to raise the σ_max_ only 13.5% (Figure 5). The study of Mollica, et al.^19^ (2004) showed a generic model with no refinement of structures (i.e. peritubular and intertubular dentin, adhesive layer based on multiple layers, and resin tags) and mesh (only 5,000 elements and 10 nodes). This might justify the differences observed on the influence of voids.

When voids were incorporated into the HL (Mp and Mpp), the peak of σ_max_ moved from the contact with the peritubular dentin to the vicinity of the voids, 2 μm above the base of the HL (Figure 6). This behavior is very similar to that found in studies with micro-tensile loading analyzing the fracture pattern in specimens of dentin sticks^24^. In these, the failure was commonly observed at the base of the HL, close to areas with poor hybridization.

Some authors^13^^,^^27^ showed that σ_max_ in the HL with poor hybridization was higher than the ultimate tensile strength of the self-etching adhesive system, even with a homogenous HL in contact with the dentin base. This issue is very important for the integrity of resin-dentin bonds over time. Lohbauer, et al.^16^ (2008) showed that resin tags do not contribute to dentin adhesion in self-etching adhesive systems, in conditions of low bond strength values. In such circumstances, this means that the adhesion of the self-etching system is solely based on the quality of the HL and on its capacity to remain bonded to adjacent structures.

The present 3-D Fe measurements showed that tensile stresses are restricted to two main sites in the dentin/adhesive interface: concentrated inside the HL, near the voids (Figure 6); and concentrated at the top of resin tags, in contact with dentinal tubule walls (Figure 6). Thus, our hypothesis can be accepted, as the σ_max_ was higher in the presence of voids. Further studies on the porous dentin/adhesive interface should be carried out, considering different degrees of bonding between the HL and the adhesive layer, as well as the intertubular dentin.

## CONCLUSIONS

Within the limitations of the present study, we can conclude that:

– The presence of voids in the hybrid layer raised the maximum principal stress in all structures of the dentin/adhesive interface;
– The increase of void content has some influence on stress. The 50% void content was able to raise the stress by 13.5% inside the HL;
– In the presence of voids, the maximum stress moved from the peritubular dentin to the HL in contact with the voids.
